# Implementation of palliative care consult Service in Hungary – integration barriers and facilitators

**DOI:** 10.1186/s12904-020-00541-0

**Published:** 2020-03-27

**Authors:** Antal T. Zemplényi, Ágnes Csikós, Marcell Csanádi, Maureen Rutten-van Mölken, Carmen Hernandez, János G. Pitter, Thomas Czypionka, Markus Kraus, Zoltán Kaló

**Affiliations:** 1 Division of Pharmacoeconomics, Faculty of Pharmacy, University of Pécs, Rákóczi street 2., Pécs, 7623 Hungary; 2 Center for Health Technology Assessment, Semmelweis University, Budapest, Hungary; 3 Syreon Research Institute, Mexikói street 65/A, Budapest, H-1142 Hungary; 4 Department of Primary Health Care, Medical School, University of Pécs, Pécs, Hungary; 5 School of Health Policy and Management, Erasmus University Rotterdam, Rotterdam, the Netherlands; 6 Institute for Medical Technology Assessment, Erasmus University Rotterdam, Rotterdam, the Netherlands; 7 Hospital Clinic de Barcelona, Institut d’Investigacions Biomèdiques August Pi i Sunyer, Universitat de Barcelona, Barcelona, Spain; 8 Center for Biomedical Network Research in Respiratory Diseases, Madrid, Majadahonda Spain; 9 Institute for Advanced Studies, Vienna, Austria

**Keywords:** Palliative care, Integrated care, SELFIE, Qualitative study

## Abstract

**Background:**

The Palliative Care Consult Service (PCCS) programme was among the first initiations in Hungary to provide palliative care for patients admitted to hospital. The PCCS team provides palliative care for mainly cancer patients and their family members and manages the patient pathway after being discharged from the hospital. The service started in 2014 with 300–400 patient visits per year. The aim of this study is to give a comprehensive overview of the PCCS programme guided by a conceptual framework designed by SELFIE (“Sustainable intEgrated chronic care modeLs for multi-morbidity: delivery, FInancing, and performancE”), a Horizon2020 funded EU project and to identify the facilitators and barriers to its wider implementation.

**Methods:**

PCCS has been selected by the SELFIE consortium for in-depth evaluation as one of the Hungarian integrated care models for persons with multi-morbidity. The qualitative analysis of the PCCS programme was based on available documents of the care provider and interviews with different stakeholders related to the programme.

**Results:**

The integrated, multidisciplinary and patient-centred approach was well-received among the patients, family members and clinical departments, as verified by the increasing number of requests for consultations. As a result of the patient pathway management across providers (e.g. from inpatient care to homecare) a higher level of coordination could be achieved in the continuity of care for seriously-ill patients. The regulatory framework has only partially been established, policies to integrate care across organizations and sectors and adequate financial mechanism to support the enhancement and sustainability of the PCCS are still missing.

**Conclusions:**

The service integration of palliative care could be implemented successfully in an academic hospital in Hungary. However, the continuation and enhancement of the programme will require further evidence on the performance of the integrated model of palliative care and a more systematic approach particularly regarding the evaluation, financing and implementation process.

## Background

Due to the growing prevalence of multi-morbidity, healthcare providers face increasing challenges with delivering appropriate care at a reasonable cost, especially for patients with palliative care needs [1–4]. Based on the results of the European Association for Palliative Care Task Force survey [5] the coverage of palliative care services has improved significantly in the past years and became more widespread in Western European countries. However, the coverage of specialist palliative care services such as hospital consultation services is very low in Central and Eastern European countries and especially low in Hungary (see Table 1). Besides home care services there were only four hospital palliative care support teams in 2015, [8], and three of these teams were not operating as part of the hospitals but as separate organizations.

**Table 1:** Coverage of specialist palliative care services in Western Europe, Central and Eastern Europe and Hungary in 2012 [6] [7]

Service	Western Europe	Central and Eastern Europe	Hungary	Recommended ratio	***Coverage of Hungary***	
	services per 100,000 inhabitants			service/100,000 inhabitants	*detected/needed*^*b*^*services; %*	
Home care team	0,4	0,21	0,69	1	*69/99; 69%*	
Inpatient palliative care services	0,35	0,14	0,13	0,5	*13/50; 26%*	
Consult services^a^	0,3	0,08	0,03		0,5	*3/50; 6%*

^a^Named in the study as hospital support team;

^b^Detected services are the actual number of services in Hungary. To calculate the demand of services needed, the population of 100,000 units is multiplied by that ratio recommended for 100,000, suggested by EAPC White Paper [7]

The need for palliative care within secondary care in Hungary has emerged due to the observed over- or undertreatment of patients and the absence of adequate management of physical and psychological symptoms. Palliative care within hospital settings is a relatively new form of care in Hungary [6]. The Palliative Care Consult Service (PCCS) programme was the first initiative in Hungary to provide palliative care within an inpatient care institution at the Clinical Centre of the University of Pécs. The Clinical Centre is an academic hospital offering a variety of services to treat the general healthcare needs of patients as well as specialized services. The institution is the only hospital in the city with 1575 beds and 27 clinical departments. The number of inpatient admissions is around 80,000 (of which 22–24% are cancer patients) per year. The academic hospital also serves as a healthcare hub for the geographic region, admitting patients transferred from other hospitals.

The aim of the PCCS programme was to establish a skilled, multidisciplinary team consisting of a palliative care physician, psychologist, and a coordinator to provide specialized consulting service for seriously-ill patients and their family members. Most of the patients with palliative care needs have multi-morbidity, as they usually suffer from multiple chronic conditions. PCCS has been selected by the SELFIE consortium for in-depth evaluation as one of Hungarian multi-morbidity integrated care models.

SELFIE (“Sustainable intEgrated chronic care modeLs for multi-morbidity: delivery, FInancing, and performancE”, Grant Agreement No. 634288), a Horizon2020 funded EU project, has established a new framework of integrated care for multi-morbidity [1, 2]. The conceptual SELFIE framework organises elements of integrated care into the six WHO components of health systems (service delivery, leadership & governance, workforce, financing, technologies & medical products, information & research), thus providing a model for a system-level approach [2]. The aim of this study is to give a comprehensive overview of the PCCS programme covering the six components of the conceptual framework for integrated care in multi-morbidity [9]. The paper also aims to share the barriers to the implementation alongside strategies that were applied to overcome them and to support the development of a formalized functional model of integrated palliative care in Hungary, as this model can serve as an example for other institutions in the Central and Eastern European Region. The detailed description of PCCS is available as SELFIE project deliverable report [10]. Further details on SELFIE can be found on the website (www.selfie2020.eu).

## Methods

The evaluation of PCCS consists of a qualitative and a quantitative research phase. The quantitative research phase (not reported in this paper) evaluates multiple patient-level outcomes related to health, patient experience and healthcare costs in a large sample of PCCS and control patients. The qualitative research described in this paper preceded the quantitative research phase and was focusing on how the PCCS programme has been initiated, conceptualized, implemented and was operating from the perspectives of various stakeholder groups. In this study the method of thick description was applied, which is a qualitative empirical research method to investigate implicit social practices, such as health care delivery. In the specific context of the SELFIE project, this formal description pertains to the general organisational structure of the programme and formal relations of the involved stakeholders. The formal description is valuable in itself, because it gives an overview of the domains and levels of integration, the individuals and organisations involved, the tools used and the processes employed.

The data collection and the evaluation was conducted by evaluators of the SELFIE program. A document analysis was performed based on various documents (e.g. official documents of the programme, grey literature and presentations) and expert information, as this was essential to obtain a basic knowledge about the programme. Most of the documents were provided by the academic hospital and some information were publicly available on the internet. More information on the thick description methodology and the documents used for the analysis is available in the SELFIE thick description report [10].

To get a deeper understanding of the features of the PCCS, interviews were conducted with different stakeholders involved in the programme. On the basis of the document analysis, a purposive sample of interviewees was defined. Interviewees had no prior relationship to the researchers and were approached personally, by email or by telephone. The main aim of the interviews was not to evaluate unique patient paths or outcomes but to understand how these stakeholder groups think about the key features of PCCS. Therefore, questions of “how” and “why” were at the centre of the interviews and the subsequent analysis of their contents. This comprehensive approach allows for a deeper understanding of what daily practice in the programme looks like. SELFIE researchers defined categories of stakeholders to be interviewed in advance: managers of the programme; initiators of the programme; representatives of the payer; professional staff (e.g. physicians, nurses); patients and informal caregivers (e.g. relatives). For each of these stakeholder groups, thematic focus areas were defined. A set of interview protocols were prepared by SELFIE, considering the different backgrounds of the individual stakeholder groups and the relevant themes to be discussed (see Table 2). This served the purpose of gaining insights into the programme from various perspectives. The included questions covered, for example, the stakeholders’ perceptions of delivery of care for persons with multiple chronic conditions, their roles and relationships to the programme and their personal views on the barriers to, and facilitators of PCCS.

**Table 2:** List of interview partners in the “Palliative Consulting Services” Programme qualitative evaluation

	Interview partner groups	Position
Managers (*N* = 4)	Manager of the programme (*N* = 2)	Medical Director of the Clinical Centre
		Head of the Department of Primary Health Care
	Initiator of the programme (*N* = 1)	Head of the Department of the Hospice-Palliative Care Department
	Representative of sponsor/payer organisations (*N* = 1)	University of Pécs, Health Insurance Department Head^a^
Physicians (*N* = 3)		Palliative care team physician
		Physician requesting consultation
		Palliative care physician in the home care
Non-physician healthcare professional (*N* = 4)		Hospice nurse and coordinator in the palliative care team
		Palliative care team psychologist
		Head nurse at a clinical department
		Head nurse at a clinical department
Informal caregivers (*N* = 2)		Relative of a patient
		Relative of a patient
Patients (*N* = 2)		Patient involved in PCCS programme
		Patient involved in PCCS programme

^a^Note that there is no specific macro-level funding to this project beyond the hospital's normal financial sources. Therefore, the interviewee was a representative of the academic hospital

Altogether 15 interviews were carried out, all face-to-face with a duration of 30 to 90 min between June and August 2016. The interviews were recorded and transcribed. Two independent researchers from the SELFIE team reviewed the transcriptions, which were then analysed using content analysis method developed by Mayring [11]. First, the main topics for each interviewee were determined. Per topic, units were selected for analysis, for example a full sentence or a paragraph. For each selected unit a paraphrasing was carried out, and then translated into English in a shorter form. The study was built through the interpretation and narrative description of the shorter form of interview paraphrases, which were structured according to the six components of the SELFIE conceptual framework. Barriers to and facilitators of the PCCS programme were summarized in each of the six components. All anonymised quotations in the study report (intended to present the stakeholders’ perspectives in their own words) were translated to English.

## Results

### Embedding the palliative care consult service programme in the Hungarian healthcare system

**Fig. 1:**
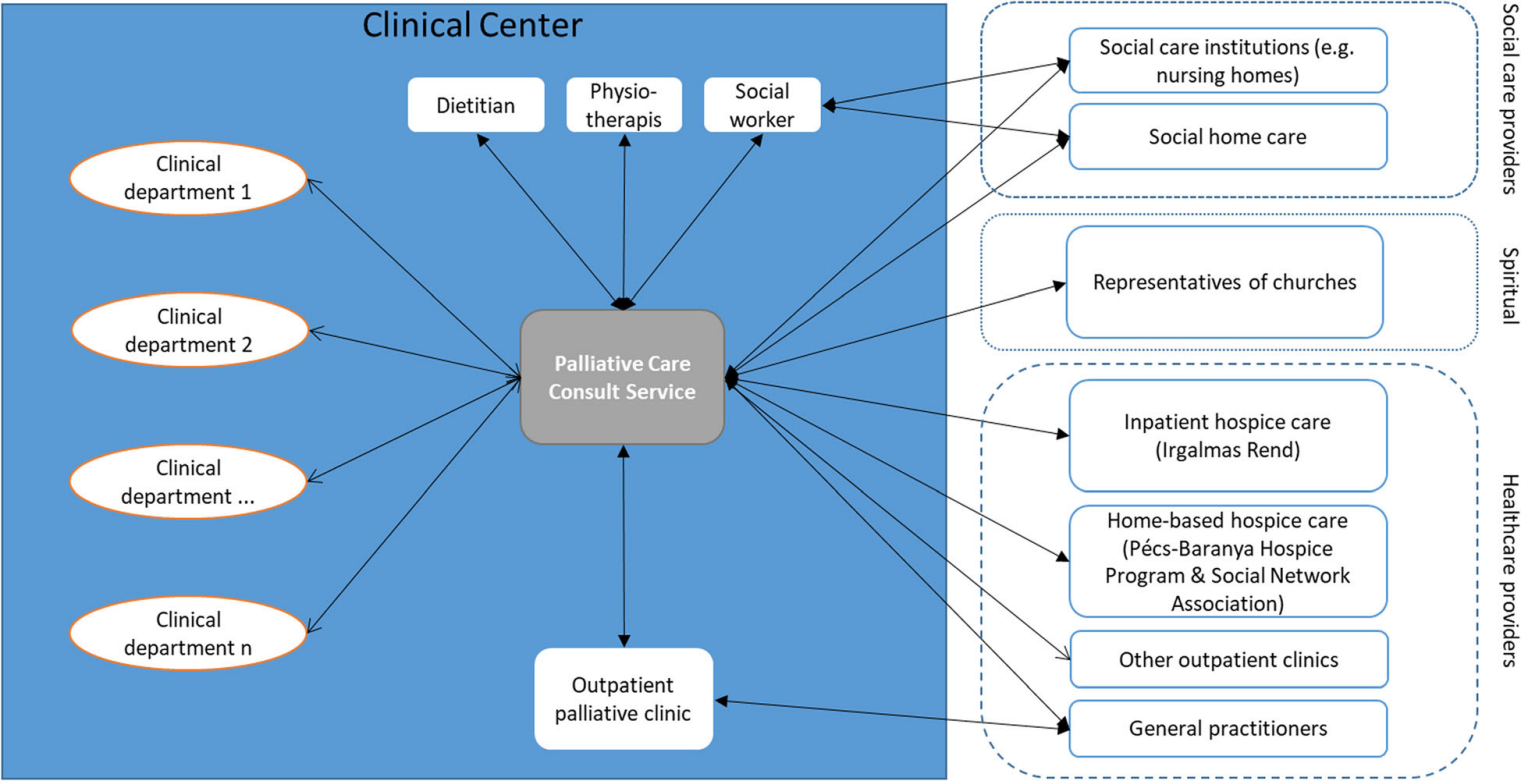
Relationship of PCCS regarding palliative care

Palliative care is provided in four main forms of service in Hungary, including inpatient hospice institutions, home hospice palliative care services, outpatient palliative care clinics and consult services. For patients living in the geographic region of the city Pécs, inpatient hospice care (organized by the Catholic Church) and the home hospice palliative service (provided by the Pécs-Baranyai Hospice Programme and Social Network Association) have been available since 2004. The Clinical Centre had set up an outpatient palliative clinic in 2012, while the most recently established provider of palliative care in Pécs was the PCCS, which started its operation in June 2013. Via the consult service, palliative care was integrated into secondary care (see Fig. 1).

### Service delivery

The target group of the programme are patients in the advanced stage of chronic progressive diseases. According to a regulation of the National Health Insurance Fund to improve complex home and institutional hospice services, 80% of the patients involved in care must be oncology patients (with the ICD-10 code “C”). For the remaining 20%, seriously-ill patients with other chronic diseases (AIDS, autoimmune diseases, progressive neurological diseases other than stroke, dementia or Alzheimer’s disease) may be involved based on the institution’s decision [2]. In the academic hospital, the PCCS’s work was mainly focused on patients with locally advanced or metastatic cancer (the proportion of oncology patients visited by the team is over 90%).

#### Process of palliative care consultations

**Fig. 2:**
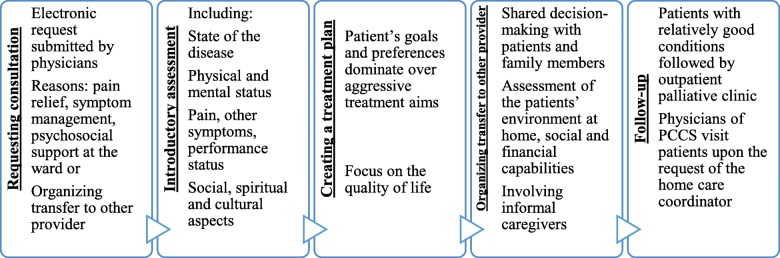
The process of palliative care within the acute care hospital

Patient care is provided by a dedicated team working closely with other hospital professionals. The task of the team is to respond quickly to the needs of the patients. For this reason, a palliative care coordinator is available 5 days a week (Monday to Friday) from 8:00–16:00 for personal consultations at any clinical departments and answers a hotline on the weekends. The process is shown in Fig. 2.

#### Requesting consultation

**Fig. 3:**
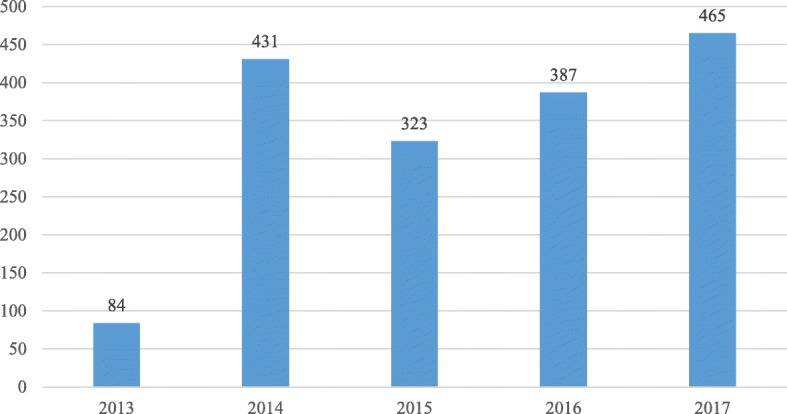
Number of palliative consultations requested, 2013–2017

**Fig. 4:**
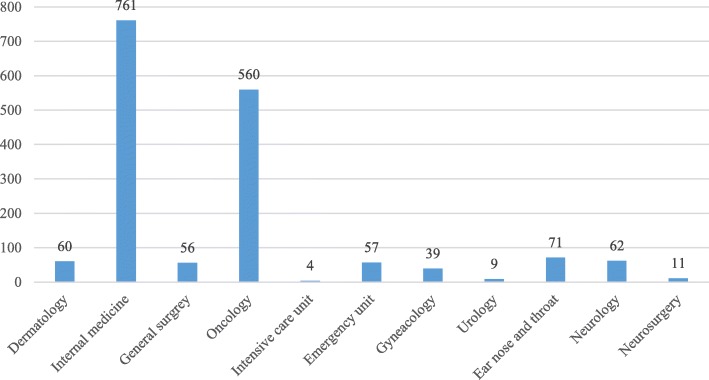
Number of palliative consultations requested by departments, 2013–2017, *n* = 1690

**Fig. 5:**
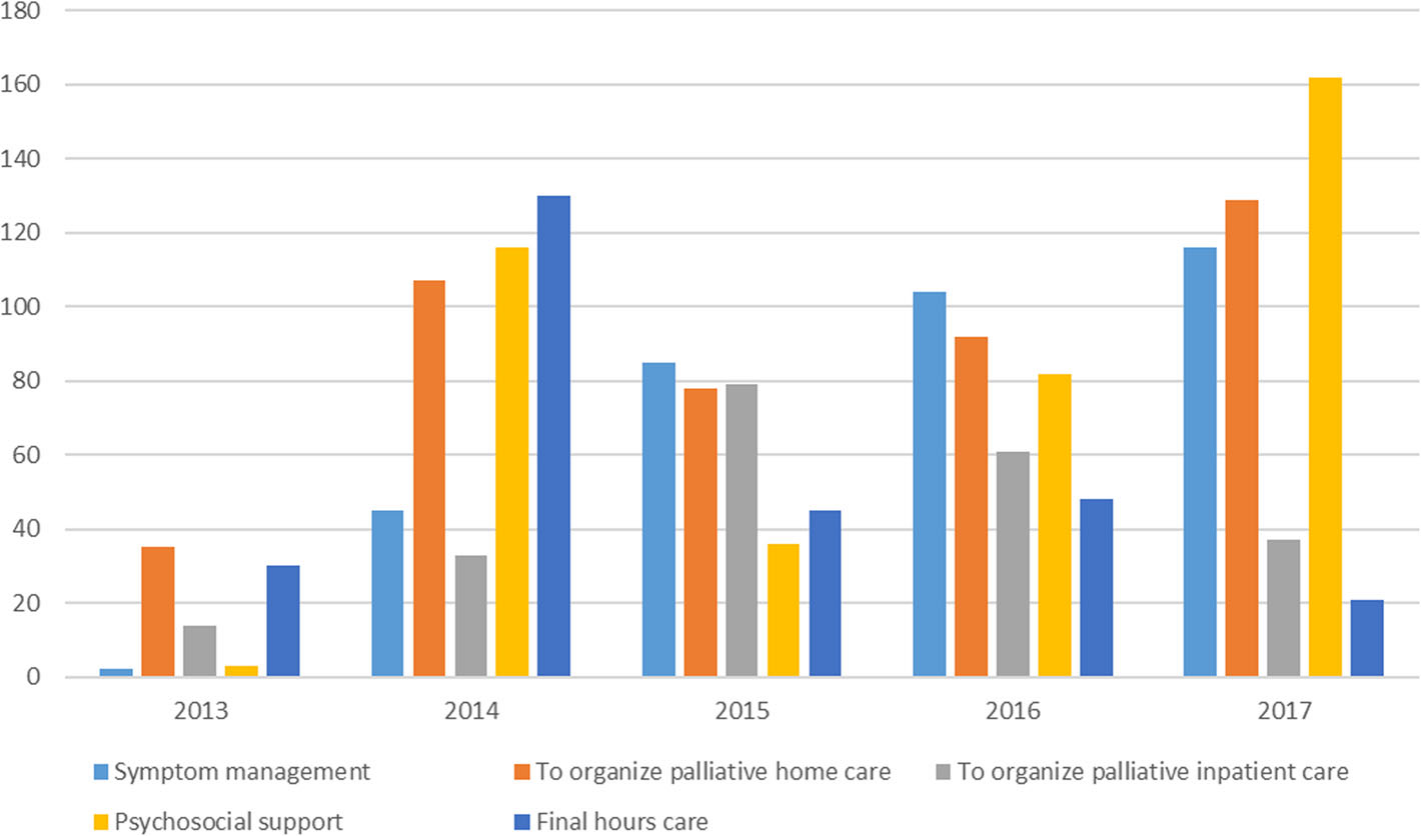
Reasons for requestion palliative consultation, 2013–2017, *n* = 1690

The team is available on request for consultations that can only be submitted by doctors from the relevant departments (see Fig. 3 and Fig. 4). The need for palliative care is often first noticed by a nurse, and then discussed with the attending physician. The main reason for requesting a consultation must be stated in the electronic referral system: 1) discharging patients to home hospice-palliative care, 2) transferring patients to inpatient hospice institution 3) providing palliative care at the ward: pain relief, management of other symptoms or 4) providing psychosocial support. A significant proportion of the requests relate to management of the discharge to home hospice-palliative care or to inpatient hospice care (see Fig. 5).

*Quotation from a patient: “Someone told me I needed a psychologist because this helpless state is making me mad…, then we talked to a nurse in the hospital and she said that there was this team who could help me. Somebody came to me maybe the next day, and said that if I wanted, they would come and tell me what it is all about. They have been with me since then (2 months ago).”*

#### Introductory assessment

The coordinator, who is a nurse in the PCCS team, visits the appropriate hospital department within 24 h of the call for consultation, assesses the performance status and needs of the patients by talking to patients and family members, reviewing available medical records, and consulting with the attending physician and nurses. The assessment report includes the disease state, physical and mental condition of the patient, pain and other symptoms, as well as social, spiritual and cultural aspects.

#### Creating a treatment plan

The involvement of patients in the treatment plan and care process is based on the preferences of the patients. In case of a need for pain relief or management of other burdensome symptoms (e.g. nausea), the coordinator involves the palliative physician in the process. The physician assesses the symptoms, discusses the expectations of patients and their family members regarding care, and listens to how well they understand the disease and its prognosis.. The physician will also consults with other specialists (e.g. oncologist) if needed to create a written, comprehensive and personalized treatment plan.

#### Organization of further care

The coordinator maintains contact with the patients and the family members. He responds to the needs of patients while they stay in the hospital. He facilitates joint decision-making with patients and family members on the further care, considering the wishes of the patients, home environment, the social and financial possibilities, and the availability of family members or informal caregivers for care. It is the task of the coordinator to share the necessary information about the treatment plan with the professionals who will provide further care.

PCCS team also provides self-management support for patients who go home. The medical aspects of self-management interventions include providing practical information to patients about care activities (e.g. cleaning the feeding tube, stoma replacement, prevention of bed-sore) or proper nutrition (e.g. to prevent constipation).

#### Follow-up on patients

Patients discharged from the academic hospital are mainly followed-up by general practitioners. Nevertheless, PCCS programme created several channels for maintaining the continuity of care. Patients with relatively good conditions are cared for by the outpatient palliative clinic (a department of the Clinical Centre), which, in cooperation with the PCCS physician, reviews the pain and other symptoms, assesses patients’ performance, and adjusts the patients’ treatment plan as needed. The same psychologist can continue the psychosocial support started in the hospital in the homes of the patients. The physicians of the PCCS team work closely with home hospice care. They visit patients at the request of the home care coordinator (e.g. to monitor pain management). When patients are transferred from the academic hospital to homecare or another facility, the palliative care team will hand over all information to the team who will be providing subsequent care. The communication, in practice, is bidirectional, and implies that home care professionals will inform the consultation service team when patients with palliative care needs are re-admitted to the hospital, and vice versa. In certain cases, palliative care patients are referred to other outpatient clinics operating outside the Clinical Centre as well.

#### Barriers and facilitators

Barriers:

- The main challenge in the implementation was the acceptance of the palliative care principles in the clinical departments, and to align the palliative care activities (such as symptom management, psychosocial support, and managing transfer to home care or final hours care) with the working procedures of the department.

Facilitators:

- In the initial phase of the programme, the manager of the PCCS team approached the heads (both medical and nursing) of the clinical departments, in which the proportion of seriously-ill patients was high and discussed how palliative care consulting service could be introduced in the care process.
- Trust in the services provided by the team could be established through regular consultation and feedback from care providers, patients and family members.
- Members of the palliative team participate in the medical and nursing education and training at the University of Pécs. In this way, they can help future physicians and nurses understand the principles of palliative care, which could influence their approach to palliative care.

### Leadership & governance

The PCCS programme was established by the Clinical Centre. The management of the Clinical Centre was very supportive in the implementation of the programme.

*Quotation from a manager: “I have initiated the submission of an application for the operation of PCCS and managed the application process. Later, I followed the development of the programme and ensured that colleagues were regularly informed at the meetings of the head nurses and physicians, as palliative care is a team effort and unfortunately we still tend not to think in terms of teams.”*

As palliative care within hospital settings was a new form of care in Hungary, the formal requirements for the operation of PCCS had to be determined and the necessary regulatory approvals had to be issued by the Office of the Chief Medical Officer (a governmental organization).

The head of the PCCS team is a palliative physician who coordinates the cooperation within the group, advises physicians in the clinical departments and keeps contact with the management of the Clinical Centre.

#### Barriers and facilitators

Barriers:

- Policies to integrate care across organizations and sectors are missing.
- Collaboration has evolved naturally across providers (Clinical Centre, homecare, hospice institution and GPs), and has not yet been formalized through contracts, organizational or structural integration.

Facilitators:

- The implementation was efficiently facilitated by the initiator of the programme, who is a well-known, acknowledged professional in the field of palliative care in Hungary and has gained experience in palliative care abroad (USA and UK).
- The management of the Clinical Centre stayed involved throughout the development of the programme.
- The programme received regulatory support as the operating licences were approved.
- In order to facilitate the partnership, palliative team members provide training for GPs on the eligibility for palliative or hospice care, communication, documentation etc. to better understand when patients need to be referred to palliative care.

### Workforce

To support the provision of palliative care in the programme, new professional roles have been created. The new role of palliative care coordinator is dedicated to the operation of the consultation service. The palliative physician and the psychologist are dedicated to provide palliative services. The core tasks, rights and duties of these new professional roles were defined by adapting international experiences to the local conditions. The activities of the coordinator, the palliative physician and the psychologist are innovative in Hungary as they provide palliative care in an inpatient environment that has not previously been performed in the Hungarian healthcare system.

*Quotation from physician: “The role of a coordinator and palliative physician have long been established in home hospice care. The PCCS is a novelty in a way that the members of the palliative consulting service go to the hospital department and provide care at the bedside. In fact, the care itself was introduced in another setting, where it had not been available earlier, although the same professionals are involved in both forms of care.”*

PCCS has 2 FTE physicians, 1 FTE psychologist and 1 FTE palliative nurse coordinator.

#### Coordinator

In addition to assessing the needs of patients and families, the role of the coordinator is to keep records of palliative care, collect data, and provide comprehensive statistics on treatment procedures. The coordinator also plays an important role in internal education and in raising awareness of palliative care. Vocational training with a special focus on palliative hospice care and patient pathway management is required. The coordinator is a full time job and the position is most often filled by a qualified nurse.

#### Palliative physician

The palliative care physician conducts a comprehensive assessment and creates a personalized symptom management plan that takes into account the needs and goals of the patient. It is also the task of the leading physician to raise awareness of the PCCS team within the facility. Palliative care requires a special license from palliative care physicians. This can be obtained through a one-year training in palliative care and pain management.

#### Psychologist

In the Hungarian healthcare system, psychologists usually provide services outside of inpatient facilities. Inpatient psychological care is therefore an innovative approach of the program. The team’s psychologist is involved in the care process if psychosocial support is needed on the ward for patients, family members, or professional caregivers. The psychologist within the PCCS team must have a degree in psychology and hospice training and experience in psychosocial care for patients living with serious illness and experience in providing support for family members.

#### Barriers and facilitators

Barriers:

- A high risk was identified (burnout, low income, lack of recognition) in terms of employee retention, which can have a strong impact on the future functioning of the team.
- There is a general shortage of physicians and nurses in Hungary and no essential changes are expected in the near future than can hinder the enhancement of the programme.

Facilitators:

- The members of the PCCS team are very committed.
- Close cooperation between providers is facilitated by the overlapping of human resources (members of the team work in parallel for more providers).

### Financing

The permit granted for the operation is a prerequisite for reimbursement arrangements, however, no contract could be concluded between the National Health Insurance Fund and the hospital for financing the PCCS. The legislation is currently not well established. Despite the lack of reimbursement from the National Healthcare Fund, the PCCS programme could be funded from a variety of sources.

One source is the DRG. Inpatient care in Hungary is reimbursed by DRGs; therefore, a certain amount of financing can be indirectly allocated to every patient visited by the PCCS in the hospital. The amount of DRG reimbursement has to cover all treatment and operating costs; therefore, not only the cost of medicines, labour (professionals), hotel services, but also the costs of diagnostic procedures, consultations and overheads. All palliative care services offered by PCCS are internal consultations for patients. An internal cross-financing method was introduced to transfer DRG revenue from clinical departments to the PCCS team. Monthly billing is based on the number of services provided by the team multiplied by the performance fee. Another source was EU funding [TÁMOP 6.2.4 A-11 / 1–2012-0065], which covered the wages of the coordinator and psychologist on the team for 3 years.

The insufficient reimbursement does not allow the full coverage of all operating costs. The Clinical Centre therefore provides additional resources.

*Quotations from a manager: “It is clear that this service is needed, but at the moment, there are many difficulties in its sustainability. It should be considered as a new form of care that requires the necessary financial resources to operate.”*

*“In order for the payer to create new codes and to extend the list of procedures, the costs should be calculated. This is, in principle, the task of the payer.”*

An interview with a health care professional in a clinical department has revealed that while the funding is not sufficient to cover all palliative care services, this does not affect the frequency or number of requests.

*Quotation from a non-physician healthcare professional: “I've never heard of anybody that we cannot call them now because it would be too expensive. I think that this has to be a low cost service, which is worth for us to be able to get the patient home. The attending physician also cares about what happens to the patient thereafter and where she or he will be transferred”.*

#### Barriers and facilitators

Barriers:

- There are major shortcomings on the macro level, regarding the financial aspects of the programme. There is no direct reimbursement for the operation of palliative consult services, and no government plan has yet been published on how to incentivize the broader deployment of such services.
- The current financial regulations do not recognize PCCS as a service to be reimbursed.
- The State Secretariat for Public Health rarely reviews the list of procedures and assigned fees. The financial management of the Clinical Centre noted that the Hungarian system lacks several codes for palliative care procedures. The available codes cover only about 60–70% of all activities performed by the team.

Facilitators:

- The government provided a secured budget (EU funding for a certain period of time) to launch this programme, which was a great incentive in the initial phase.
- Internal budget transfers are used to provide funding for the programme.

### Technologies & medical products

The PCCS programme has not prioritized the improvement and enhanced use of information and communication technology (ICT) systems. However, the hospital information system (eMedSolution) at the Clinical Centre has been changed to support the palliative care requests and documentation within the institution. A special module was developed to request consultation from the team and to keep records of the electronic documentation.

The system contains information on the reasons for the requested consultations, recommendations and information on the further care process of patients, and specific information on 1) the patients’ ability to self-care (e.g. Karnofsky Performance Status Scale) and 2) the type and level of pain on a specific symptoms scale (e.g. Numeric Rating Scale).

Some of the stakeholders (nurses, managers, informal caregivers) have expressed their needs for new IT applications (e.g. remote monitoring, improved care information system and video consultation) to be used in the care process.

*Quotation from an informal caregiver: In my opinion, … that solution might be good for us…, when the patient occasionally makes a recording or writes a note, which is accessible to the doctor. The point is that the doctor should have regular information on the patient he is treating, and can look at the patient's records, and if necessary, call or ask if she or he is okay, if everything goes fine…*

#### Barriers and facilitators

Barriers:

- Home based hospice care only has paper-based documentation, which is inefficient.
- A shortcoming of the current system is that the documentation cannot be tracked electronically by providers, especially outside the hospital. Therefore, no IT support can currently be used to ensure continuity of care. After the transfer of patients to another service provider (e.g. homecare, hospice), the patients are followed-up by telephone and personal visits.

Facilitators:

- The operation of the PCCS programme was supported through the establishment of a specific electronic referral system.

### Information & research/monitoring

The programme conducts annual retrospective anonymous analyses of PCCS activities. Indicators include: 1) number of requested consultations, 2) reasons for referral, 3) time data for entering and leaving the palliative care process, 4) key symptoms at enrolment.

Although no quality assurance system has been set up within the program yet, the team members take the time and effort to share the indicators of their activities. The data from the analyses are compared with the previous year’s results, and the information is presented in internal meetings with clinical directors and senior nurses to discuss the challenges and opportunities for further improvement.

A formalized evaluation taking into account the health outcomes, the costs and the use of services and experience with care has not been carried out so far. There are informal ways of giving feedback on satisfaction with care, for example in newspaper articles or in other natural ways in the form of acknowledgments (cards, letters) given by patients or family members.

The process indicators relate more to the operation of the programme than to the impact on health, costs, or experience. Programme management recognized the need for a proper assessment of impacts and results in a more scientific approach.

*Quotation from a manager: “I think it was a pilot program, and I think we are now in the evaluation period. This is necessary to standardize the semi-formalized or non-formalized elements of the programme, and to describe them as a model that could be of help to any other institution in the country where they want to start such a program.”*

#### Barriers and facilitators

Barriers:

- In addition to data on the use of hospital resources (length of stay, readmissions, emergency visits etc.), limited data are available at the individual level (electronic documentation) to assess health outcomes, patient experiences and costs.

Facilitators:

- A prospective data collection has been initiated to obtain information that can be used for policy and research purposes.

## Discussion

The role of palliative care consultation teams is to provide on-demand services within the hospital [12]. In this qualitative study, the characteristics of the PCCS programme were examined in a structured manner. The impressions gained through the interviews have helped to understand the functioning of the program, the acceptance of the integrated, multidisciplinary and patient-centred palliative approach in the Hungarian healthcare system, and what challenges the programme has had to face.

Person-centeredness and a holistic approach characterize the PCCS program. The focus of services is mainly on the needs of patients and family members. Decisions are therefore made by directly involving them. Tailored care is provided by offering alternative treatment options. In this regard, patients’ wishes rather than aggressive medical treatment aims dominate. Informal caregivers are also involved in decision-making and education on self-management interventions. In addition to the palliative care concept, the PCCS programme supports continuity by coordinating care between providers and managing patient pathways (e.g. transferring patients form inpatient care to homecare). A recent multicentre cohort study in France showed that there is still late referral to palliative care and that the “on request” model which originally characterized these services is gradually being exchanged for the integrated palliative care model [12]. Several studies suggest that even after one visit with the palliative care team in inpatient setting, there are improvements in physical and psychological symptoms, quality of life, as well as patient satisfaction with care [13–19]. This shift towards integrated palliative care is of great importance.

The PCCS program was originally a bottom-up initiative that addressed the need for on-site palliative care and better management of patient pathways. The programme was introduced by a recognized specialist in palliative care in Hungary, who gained experience in the United Kingdom and the United States and transferred knowledge from abroad [20]. The PCCS sought to match its service portfolio to the needs defined by the clinical departments. The success of collaboration depended mainly on the attitude of the individual attending physician towards the concept of palliative care. In many cases, the head nurse of the department also became the primary contact for the team, as in Hungary, palliative care is currently better embedded in nurse training than in the medical education of physicians.

Healthcare decision-makers in Hungary expressed their commitment to set-up consultation services by allocating funding (EU funds) to start the programme. Without this incentive, it would almost certainly not have started. However, no financial support was granted to sustain the operation after the implementation period, and no plan has yet been published on how to create incentives for the wider deployment of PCCS. The continuation of the PCCS programme was a local decision by the management of the Clinical Centre, which continued to fund the programme from internal budget. The enhancement of the programme requires further evidence on the costs and outcomes of this service.

An earlier retrospective cost analysis in the US demonstrated that palliative care was associated with significantly lower impatient costs compared with usual care. Their study implicated that improved patient outcomes due to palliative care would be a cost and quality incentive for healthcare providers to initiate and elaborate palliative care programs [4]. These preliminary results of cost savings were backed by more recent comprehensive literature reviews of palliative care interventions, which demonstrated that palliative care was less costly relative to comparator groups, and in most cases, the difference in cost was statistically significant [2, 21]. Studies show that the rate of hospital cost savings with the involvement of palliative care ranges from 9 to 32% [3, 4, 22–26]. Within the SELFIE project, an empirical evaluation of the PCCS is being conducted that includes multiple outcomes, as divided into a triple aim: improvement in health and well-being, experience with care, and costs.

The PCCS program was originally a bottom-up initiative that addressed the need for on-site palliative care and better management of patient pathways. The programme was introduced by a recognized specialist in palliative care in Hungary, who gained experience in the United Kingdom and the United States and transferred knowledge from abroad [20]. The PCCS sought to match its service portfolio to the needs defined by the clinical departments. The success of collaboration depended mainly on the attitude of the individual attending physician towards the concept of palliative care.

The key elements for the successful implementation can be summarized as follows: 1) the expertise and commitment of the initiator, who could introduce a proper service delivery process; 2) support of the institutional management regarding financing the programme; 3) efficient communication with and ongoing involvement of the clinical departments in planning care process; 4) establishment of a new professional role (coordinator) to manage the patient pathway across departments and providers; 5) EU funding, which was a great incentive in the early phase of the programme. The key barriers are related to the inappropriate regulatory framework: policies to integrate care across organizations and sectors and adequate financial mechanism to reimburse the operation of the programme are still missing. There are also serious concerns regarding staff retention in healthcare, especially in palliative care, and the lagging behind in IT development.

A natural limitation of our study is determined by its qualitative design - the present research was unable to quantify the added value of PCCS for the health system in Hungary. The insights presented in this study are based on a limited number of personal views and experiences and therefore not every aspect of the programme could be examined. However, we believe that the in-depth understanding of various stakeholder perspectives, facilitators and barriers identified in our analysis can serve as guidance when designing such form of palliative care and creating the adequate conditions for its sustainable operation (especially in Central and Eastern European countries).

## Conclusion

The service delivery process of palliative care consult team could be successfully introduced in an academic centre in Hungary; however, relevant health policy regulations and financial schemes to support palliative care consultation services are still missing. The implementation of integrated care service delivery is feasible on institutional levels but the sustainability and enhancement of such programmes require a more systematic approach. The EU funded SELFIE project aims to address these constraints by developing a performance-monitoring tool to evaluate integrated care models, providing financing schemes with adequate incentives to implement integrated care and developing implementation strategies.

## Data Availability

The data that support the findings of this study are available from the University of Pécs but restrictions apply to the availability of these data, which were used under license for the current study, and so are not publicly available. Data are however available from the authors upon reasonable request and with permission of the University of Pécs.

## Abbreviations

ICT: Information and communication technology
PCCS: Palliative Care Consulting Service
SELFIE: Sustainable Integrated Chronic Care Models For Multi-Morbidity: Delivery, Financing, And Performance
